# Tumor Immune Microenvironment in Intrahepatic Cholangiocarcinoma: Regulatory Mechanisms, Functions, and Therapeutic Implications

**DOI:** 10.3390/cancers16203542

**Published:** 2024-10-20

**Authors:** Angela Dalia Ricci, Alessandro Rizzo, Annalisa Schirizzi, Rosalba D’Alessandro, Giorgio Frega, Giovanni Brandi, Endrit Shahini, Raffaele Cozzolongo, Claudio Lotesoriere, Gianluigi Giannelli

**Affiliations:** 1 Medical Oncology Unit, National Institute of Gastroenterology, IRCCS “S. de Bellis” Research Hospital, 70013 Castellana Grotte, Italy; 2 S.S.D. C.O.r.O. Bed Management Presa in Carico, TDM, IRCCS Istituto Tumori “Giovanni Paolo II”, Viale Orazio Flacco 65, 70124 Bari, Italy; 3 Laboratory of Experimental Oncology, National Institute of Gastroenterology, IRCCS “S. de Bellis” Research Hospital, 70013 Castellana Grotte, Italy; 4 Osteoncology, Soft Tissue and Bone Sarcomas, Innovative Therapy Unit, IRCCS Istituto Ortopedico Rizzoli, 40136 Bologna, Italy; 5 Medical Oncology, IRCCS Azienda Ospedaliero-Universitaria di Bologna, 40138 Bologna, Italy; 6 Department of Medical and Surgical Sciences, University of Bologna, 40138 Bologna, Italy; 7 Gastroenterology Unit, National Institute of Gastroenterology-IRCCS “Saverio de Bellis”, 70013 Castellana Grotte, Italy; 8 Scientific Direction, National Institute of Gastroenterology, IRCCS “S. de Bellis” Research Hospital, 70013 Castellana Grotte, Italy; gianluigi.giannelli@irccsdebellis.it

**Keywords:** cholangiocarcinoma, biliary tract cancer, immunotherapy, tumor microenvironment, macrophages, immune checkpoint inhibitors

## Abstract

Treatment options for intrahepatic cholangiocarcinoma (iCCA), a highly malignant tumor with poor prognosis, are limited. Recent developments in immunotherapy and immune checkpoint inhibitors (ICIs) have offered new hope for treating iCCA. However, several issues remain, including the identification of reliable biomarkers of response to ICIs and immune-based combinations. Tumor immune microenvironment (TIME) of these hepatobiliary tumors has been evaluated and is under assessment in this setting in order to boost the efficacy of ICIs and to convert these immunologically “cold” tumors to “hot” tumors. Herein, the review TIME of ICCA and its critical function in immunotherapy. Moreover, this paper also discusses potential avenues for future research, including novel targets for immunotherapy and emerging treatment plans aimed to increase the effectiveness of immunotherapy and survival rates for iCCA patients.

## Simple Summary

Treatment options for intrahepatic cholangiocarcinoma (iCCA), a highly malignant tumor with poor prognosis, are limited. Recent developments in immunotherapy and immune checkpoint inhibitors (ICIs) have offered new hope for treating iCCA. However, several issues remain, including the identification of reliable biomarkers of response to ICIs and immune-based combinations. Tumor immune microenvironment (TIME) of these hepatobiliary tumors has been evaluated and is under assessment in order to boost the efficacy of ICIs and to convert these immunologically “cold” tumors to “hot” tumors. Herein, we examine the role of iCCA TIME, highlighting its mechanisms, current applications and challenges, and future research directions.

## 1. Introduction

Over the past few decades, there has been an increase in the incidence of intrahepatic cholangiocarcinoma (iCCA), a malignant tumor arising from the intrahepatic secondary bile ducts and their branches [1,2,3,4]. For most patients, the lack of early symptoms results in diagnosis at an advanced stage, and due to the complexity of biology and high heterogeneity of iCCA, traditional treatments like surgical resection, chemotherapy, and radiotherapy are frequently ineffective [5,6,7,8]. As a result, the 5-year survival rate following radical surgical resection is about 30% [9,10].

The development of cancer immunotherapy in the past twenty years has improved clinical outcome for a variety of hematological and solid tumors [11,12,13,14,15,16]. Since anticancer agents targeting programmed cell death protein 1 (PD-1), programmed death-ligand 1 (PD-L1), cytotoxic T-lymphocyte antigen 4 (CTLA-4), lymphocyte activation gene 3 (LAG-3), T-cell immunoglobulin domain and mucin domain 3 (TIM-3), and others have reported important results in phase I to III clinical trials [17,18,19]. Immunotherapy fights tumor cells by stimulating and boosting the patient’s immune system, and ICIs represent a fundamental avenue of research in this setting. These agents also offer new hope for biliary tract cancers, including iCCA [20,21,22]. However, unlike other solid tumors, iCCA has a different response to immunotherapy for several possible reasons, including distinct tumor immune microenvironment (TIME) and immune escape mechanisms [23,24,25]. Indeed, several issues remain, including the identification of reliable biomarkers of response to ICIs and immune-based combinations [26]. TIME of these hepatobiliary tumors has been evaluated and is under assessment in order to enhance the efficacy of ICIs and to convert these immunologically “cold” tumors to “hot” tumors [27,28]. However, despite a strong biological rationale and some preclinical reports, TIME is not part of everyday clinical practice in iCCA immunotherapy, and further efforts are needed.

In the current Review, we examine the role of iCCA TIME, highlighting its mechanisms, current applications, challenges, and future directions.

## 2. The Tumor Immune Microenvironment of Intrahepatic Cholangiocarcinoma

**Figure 1:**
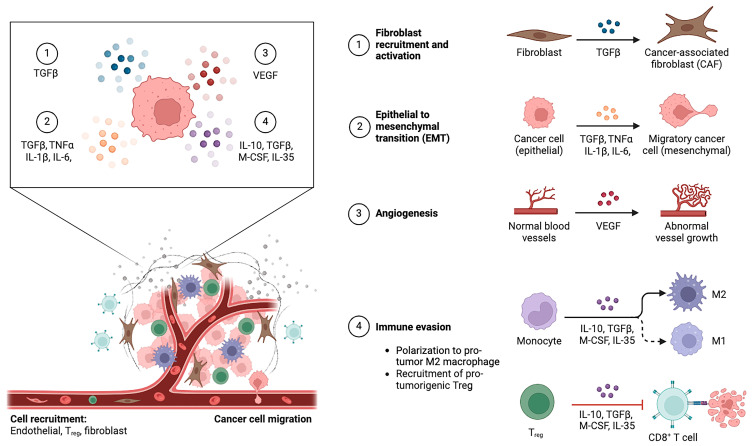
Overview of cancer-associated changes in tumor immune microenvironment.

TIME of iCCA comprises a various range of cellular and non-cellular components that are extremely dynamic and intricate [29,30]. Immune cells, cancer cells, fibroblasts, and endothelial cells are among the cellular components of TIME, and these cells interact with each other through a variety of cytokines, chemotactic factors, and growth factors that influence tumor growth and invasion (Figure 1) [31]. Important roles are also played by non-cellular components such as the extracellular matrix (ECM) and soluble signaling mediators like Transforming Growth Factor β (TGF-β), a profibrogenic cytokine converting fibroblasts in myofibroblasts [32,33], and Platelet-Derived Growth Factor (PDGF), a pro-angiogenic and pro-fibrotic molecule [34]. The mutual interaction between tumor cells and their microenvironment results in the activation of Notch signaling pathways involved in the initiation and progression of intrahepatic cholangiocarcinoma. In particular, Notch was shown to be essential for the regulation of angiogenic sprouting and tumor vessel growth in conjunction with Vascular endothelial growth factor A (VEGF-A) [35]. Thus, the proliferating fibrous stroma within the iCCA TIME is highly reactive and has pro-tumorigenic, pro-angiogenic, and immunosuppressive properties that impair the effectiveness of anticancer therapies [36]. Thus, effective immunotherapeutic strategies would require a deep understanding of the cellular and non-cellular components of iCCA TIME and their interactions.

### 2.1. Cellular Elements of Tumor Immune Microenvironment

#### 2.1.1. Cancer-Associated Fibroblasts

A number of studies have shown that iCCA is characterized by a prominent desmoplastic stroma, which is mainly enriched in cancer-associated fibroblasts (CAFs). CAFs are characterized by strong expression of alpha-smooth muscle actin (alpha-SMA), driven by TGF-β, and widely interact with iCCA cells and immune cells. This enhances the malignant phenotype and promotes tumor fibrosis and progression through several mechanisms, leading to therapy resistance [37,38]. Indeed, CAFs are involved in promoting lymphangiogenesis and angiogenesis and in the formation of a pro-inflammatory tumor microenvironment by secreting growth factors, cytokines, and several chemotactic factors, such as fibroblast growth factor (FGF), vascular endothelial growth factor (VEGF), and C-X-C motif chemokine ligand 12 (CXCL12) [38,39]. Furthermore, CAFs increase tumor invasiveness and growth by altering the ECM and releasing matrix metalloproteinases (MMPs) and other stromal proteins [40]. The remodeled and altered ECM acts as a barrier, limiting the infiltration of immune cells and is associated with reduced cytotoxic T-cell infiltration, increased tumor-associated macrophage (TAM) infiltration, and a poorer prognosis [41]. Hepatic stellate cells, portal fibroblasts, bone marrow mesenchymal stem cells, and fibroblasts derived from epithelial-mesenchymal transition and endothelial-mesenchymal transition are among the cells from which CAFs are activated in iCCA [42]. Recent research has shown that inducing apoptosis in CAFs with pharmacological agents, such as the BH3 mimetic navitoclax, can dramatically reduce tumor growth and prevent metastasis and lymphovascular invasion [43,44]. Moreover, CAFs have complex interactions with both immune and tumor cells, particularly through the CCL2-STAT3 signaling pathway [45]. These cells also exhibit immunosuppressive properties, including the inhibition of T-cell proliferation and function and the suppression of adaptive immune responses, which favor tumor immune escape [46]. Thus, studies and treatment strategies that focus on CAFs and their unique signaling pathways are key to advancing the knowledge of tumor biology and opening the door to novel immunotherapeutic advancements for iCCA.

#### 2.1.2. Tumor-Associated Macrophages

The M2-type of tumor-associated macrophages (TAMs) is especially important for TIME [47]. Due to their primary involvement in angiogenesis and tissue repair, these macrophages play a role in the development of tumors [48]. M2-type TAMs secrete proinflammatory and pro-angiogenic factors, such as TNF-α, IL-6, TGF-β, and VEGF-A, which exacerbate tumor invasiveness and metastasis. In particular, these macrophages actively alter TIME through their interactions with cancer cells by promoting tumor growth and local invasiveness [49]. Additionally, by expressing inhibitory receptors like PD-1 and Siglec-10, which bind to ligands on tumor cells and facilitate tumor immune escape, M2-type TAMs inhibit the immune system’s phagocytic functions [50,51].

Recent reports have suggested a strong correlation between iCCA aggressiveness, increased Treg infiltration, and a grim prognosis when there is a high density of M2-type TAMs [52]. Thus, novel therapeutic approaches are being developed to fight this immunosuppressive state; these strategies include repolarizing M2-type TAMs to M1-type by activating the Notch pathway or employing interferon gamma (IFN-γ), which improves the TAMs’ antitumor properties [53]. Recent research has examined chimeric antigen receptor macrophages (CAR-M), which are genetically modified macrophages that express particular tumor antigen receptors [54]. Several reports have suggested that the effectiveness of immunotherapy could be increased when these CAR-M cells target and phagocytose tumor cells in addition to triggering T-cell responses [55,56,57]. This novel and multidisciplinary strategy opens up new treatment options for iCCA and has great promise to overcome the drawbacks of traditional immunotherapy.

#### 2.1.3. Tumor-Associated Neutrophils

Tumor-associated neutrohils (TANs) play a key role in the TIME, despite their effect on the prognosis of patients, which is still to be defined [58]. While high TAN infiltration has been associated with poor prognosis, recent research suggests that high neutrophil infiltration may be associated with prolonged survival [59]. This disparity may be due to the different TAN phenotypes and behaviors within TIME, and some reports have highlighted that the two polarized TAN phenotypes, N1 and N2, have distinct functions at different stages of tumor progression: N1 shows antitumor characteristics in early stages, while N2 has immunosuppressive properties that promote tumor growth and metastasis in later stages [60]. The primary regulators of TAN function and polarization are TAM-secreted factors such as TGF-β and tumor cells [61]. By up-regulating factors such as arginase 1 (ARG1) and CCL2, TGF-β promotes tumor growth and the potential for metastasis by facilitating the conversion of N1-type TANs to N2-type [62]. Moreover, the function of TANs in boosting the metastatic potential of circulating tumor cells has been revealed by single-cell RNA sequencing [63]. Through the release of growth factors and cytokines, these N2-type TANs promote tumor immune escape by inhibiting effector T-cell function and stimulating angiogenesis [64]. Therapeutically, TAN polarization can be effectively modified and CD8+ T cell antitumor activity increased by exposing tumor-specific antigens with radiotherapy or TGF-β receptor inhibitors combined with granulocyte colony stimulating factor [65]. Based on these premises, neutrophil-based therapeutic approaches are set to become a rapidly evolving area of cancer therapy.

#### 2.1.4. Myeloid-Derived Suppressor Cells

Myeloid-derived suppressor cells (MDSCs) are a subset of immature myeloid cells found in the TIME that mainly stimulate the growth and metastasis of tumors by inhibiting the actions of both innate and adaptive immune systems, with a specific focus on CD8+ T cells [66]. Their accumulation in TIME promotes tumor angiogenesis by secreting pro-angiogenic factors like VEGF, which inhibits T cell activation and function [67]. Moreover, by overexpressing PD-L1, which binds to PD-1 on T cells, MDSCs aid in the depletion of T cells and facilitate immune escape [68].

Due to their variety of functions, MDSCs are important potential targets for immunotherapy [69]. Research shows that MDSCs may prevent T cell activation in tumor patients by catabolizing or depleting cysteine and arginine, which inhibit T cell metabolism and lower the expression of homing receptors on T cell surfaces [70]. MDSC levels in the blood are markedly higher in iCCA patients, and they are highly correlated with the prognosis and severity of the disease [71]. In preclinical animal studies, strategies that target MDSCs, such as lowering their population by using anti-granulocyte differentiation antigen-1-specific antibodies, have shown promise in preventing tumor growth [72,73]. Additionally, decreasing the activation and aggregation of MDSCs at the tumor site may improve immunotherapy’s efficacy and provide iCCA patients with more effective treatment options [74].

#### 2.1.5. Natural Killers

As essential members of the innate immune system, natural killers (NKs) have been shown to have strong antitumor activity, and in the absence of previous antigen exposure, these cells are non-specifically able to identify and attack cancer cells [75,76]. NKs comprise approximately 30–40% of all lymphocytes in the liver, and they mainly use cytotoxic factors such as FasL and TRAIL, as well as cytokines like TNF-α and IFN-γ, to achieve their antitumor effects [77,78]. Furthermore, NKs play a critical role in controlling tumor cell growth and metastasis as well as inducing apoptosis. According to recent research, increasing the number or functionality of NKs may effectively halt the advancement of iCCA, establishing them as possible targets for treatment [79,80].

Some reports have suggested a strong correlation between the prognosis of iCCA patients and the expression of the NK activation receptor NKG2D [81]. Patients with higher expression levels of NKG2D typically have better prognoses, probably because NKG2D helps NKs to identify and destroy cancer cells. Furthermore, by triggering apoptosis in tumor cells and by regulating adaptive immune responses, NKs show promise in precision immunotherapy by lowering off-target effects through cytokine release [82]. The promise of NKs-based immunotherapeutic strategies has been highlighted by experimental studies that suggest a significant inhibition of tumor growth following the infusion of human NKs into a xenograft mouse model by using the human CCA cell line HuCCT-1 [83,84].

#### 2.1.6. Tumor-Infiltrating Lymphocytes (TILs)

Tumor-Infiltrating Lymphocytes (TILs) are immune cells that directly participate in the immune response against tumor cells [82]. They are composed of different subpopulations, such as CD8+ cytotoxic T cells and CD20+ B cells, and they play a complex immunomodulatory role within the TIME [85]. Research has indicated that TILs are able to identify tumor antigens and trigger immune responses against tumors [86,87]. Through encouraging TIL apopoptosis, certain signaling pathways, such as TGF-β, PD-1/PD-L1, and Wnt/β-catenin, boost tumor immune escape. While extensive CD4+ T cell infiltration is correlated with longer overall and recurrence-free survival, high levels of CD8+ TILs are generally linked to improved patient survival [88].

There is a great deal of variation in the TIME in terms of TIL types and functions. For example, CD4+ T cells are more frequently located in the periphery of the tumor, whereas CD8+ TILs usually concentrate in the inner regions of the tumor [89,90]. Because they are unable to produce the proteases necessary to break down the extracellular matrix, CD8+ T cells are unable to permeate dense tumor tissue, although they do tend to concentrate at sparse fibrous junctions and the collagen-rich peripheral matrix [91]. Furthermore, the density and orientation of the matrix structures may have an impact on the distribution of TILs within TIME.

Tumor-associated antigens (TAAs) found in iCCA may be targets for cancer vaccines that seek to boost TILs’ immune responses [92]. The T cell populations in iCCA vary, according to recent transcriptome studies. Notably, CD8+ TILs from highly heterogeneous tumors show reduced cytotoxic potential [93,94]. Furthermore, a distribution that is associated with a worse prognosis was noted: CD8+ TILs are mainly found at the invasive margins of tumors, whereas Tregs and IL-17-positive immune cells are more common inside the tumor [95,96]. In addition, FOXP3, a transcription factor that is primarily overexpressed by Tregs, is linked to elevated CTLA-4 expression and may be a sign of chemoresistance and tumor recurrence. Tregs in TIME alter the phenotypic of the cells by triggering CCL2, which causes the release of immunosuppressive substances such as IL-10 and TGF-β [97]. This encourages tumor immune evasion and inhibits effector T-cell activity. To develop more potent immunotherapeutic strategies, a thorough comprehension of the functional and regulatory mechanisms targeting TILs is essential, especially in overcoming the immunosuppressive state in iCCA.

#### 2.1.7. Dendritic Cells

As specialized antigen-presenting cells, dendritic cells (DCs) are essential elements of the TIME of iCCA [98]. These cells have excellent antigen-uptake, processing, and presentation skills. While mature DCs are good at activating initial T and B cells—which are essential for starting, regulating, and maintaining the immune response—mature DCs are highly capable of migrating [99]. Mature DCs (CD83+) are primarily located at the invasive tumor margins in iCCA, while a considerable number of immature DCs (CD1a+) are found inside the tumor [100,101]. A favorable prognosis is closely associated with the presence of mature DCs, which positively correlate with the quantity of CD4+/CD8+ cells in TIME. This enhances T cell activation and the antitumor immune response [102].

On the other hand, TIME has a significantly lower DC density, which may be due to a decrease in the amounts of chemokines (like CCL4 and CCL5) that attract DCs [103]. These chemokines are usually generated by lymphocytes, including NKs. Increased TIME concentrations of TGF-β, adenosine, IL-10, and hypoxia can induce DCs to exhibit an immunosuppressive phenotype [104,105]. This phenotype promotes the recruitment of MDSCs, M2-type TAMs, N2-type TANs, and the polarization of Tregs in addition to counteracting the tumor-fighting effects of effector T cells through immune checkpoint ligands and causing T cell depletion [106,107]. It does this by secreting IL-6 and triggering the production of TGF-β.

Based on these premises, there are several opportunities for tumor vaccine developments, according to the characteristics of DCs [108]. Clinical trials have tested tumor vaccine technologies for some solid tumors, including prostate cancer and melanoma [109,110,111,112]. These technologies take advantage of the properties of DCs to load tumor antigens and to activate immune effector cells. Enzyme-loaded DCs have enhanced CD3+ lymphocyte infiltration in iCCA animal models, which effectively inhibits tumor growth and metastasis [113,114]. These results suggest that improving DCs’ capacity for immune activation and antigen presentation may increase the effectiveness of immunotherapy against iCCA, especially when used in combination with other immunomodulatory techniques.

### 2.2. Non-Cellular Elements of Tumor Immune Microenvironment

#### 2.2.1. Extracellular Matrix

In order to facilitate the structural remodeling of tumor tissues through the release of essential components like MMPs, osteoprotegerin, junctional protein-C, and osteonectin, extensive reorganization of the ECM is essential for tumorigenesis and progression [115]. The growth of tumors, increased lymph node metastasis, and overall survival are all strongly correlated with these components’ expression. Through the activation of the RAS-RAF-MEK-ERK and Wnt/β-catenin signaling pathways, elevated osteonectin expression in TIME of iCCA correlates significantly with tumor size, local and distant invasion, and advanced cancer stages [116,117]. Furthermore, osteonectin promotes T cell survival and NK development, highlighting the diverse functions of the ECM in controlling the tumor immune milieu.

Increased ECM stiffness is found to be a characteristic of tumor progression in iCCA studies [118,119,120]. In addition to stimulating cell proliferation, survival, migration, and differentiation, which in turn drives tumor progression, ECM’s increased stiffness also affects tumor behavior through the extracellular vesicles that contain proteins, lipids, and nucleic acids secreted by tumor cells [121]. These vesicles represent a complex and important mechanism in the progression of tumors, as they contribute to immune regulation, tumor angiogenesis, and the reconstitution of TIME [122]. Targeting ECM may have therapeutic benefits, and a particular goal of this research is to improve the prognosis and outcomes of iCCA patients by modifying important ECM elements.

#### 2.2.2. Chemokines

A subclass of small-molecule cytokines called chemokines controls the chemotactic migration of leukocytes, including T lymphocytes, macrophages, and monocytes [123,124]. They are essential for the development of iCCAs because chemokines influence the migration, invasion, and immune escape of cancer cells [125]. Chemokines that facilitate the necessary oxygen and nutrient supply to tumors, such as CCL11, CCL2, CXCL1-CXCL3, CXCL5-CXCL6, CXCL8-CXCL12, and CCL16, either directly or indirectly, promote angiogenesis [126]. To be more precise, angiotensin II and TGF-β both negatively and positively regulate CXCL12 in iCCA, while CXCR4 expression is encouraged by TAM-secreted TNF-α. The activation of multiple signaling pathways, such as the Wnt/β-catenin, extracellular signal-regulated kinase (ERK1/2), and phosphatidylinositol 3-kinase/protein kinase B (PI3K/AKT) pathways, is facilitated by these chemokines through their specific receptor interactions [127,128,129].

For example, fibroblast activating protein (FAP)-induced CCL2 controls the migration of MDSCs and macrophages, while CXCL5 functions as a chemokine for neutrophils [130]. Chemokine-targeting clinical trials are currently being conducted to investigate the clinical utility of chemokines in oncology treatment, ranging from hematological malignancies and breast cancer to hepatobiliary tumors like iCCA [131,132]. The results of these studies may provide therapeutic targets and important biological insights for the development of novel immunotherapeutic approaches to treat iCCA [133,134].

#### 2.2.3. Extracellular Vesicles

Extracellular Vesicles (EVs) are microscopic membrane structures that serve as messengers between cells and are essential for TIME [135]. These vesicles can be divided into three sizes based on their diameter: exosomes (30–100 nm in diameter), microvesicles (0.1–1 μm), and apoptotic bodies (greater than 1 μm in diameter). The latter are usually removed by phagocytosis shortly after their release [136]. Proteins, lipids, messenger RNAs (mRNAs), microRNAs (miRNAs), long noncoding RNAs (lnRNAs), and circular RNAs (circRNAs) are the constituents of EVs, and the lipid bilayer of the vesicles protects these components from enzymatic degradation [137].

EVs in iCCA stimulate mesenchymal stem cell migration and the release of different cytokines and chemokines that aid in the growth of the tumor [138]. For instance, α-smooth muscle actin and other markers can be expressed by EVs derived from iCCAs [139]. Additionally, EVs are thought to be possible new therapeutic targets because they promote interactions between iCCA cells. Research has suggested that plasminogen activator inhibitor 2 (PAI-2) secretion is enhanced when mRNA-15a is downregulated in CAFs, which in turn promotes tumor cell migration [140,141]. The therapeutic potential of miRNAs has been demonstrated in animal models through the infusion of miRNA-195-enriched EVs, which has been shown to reduce tumor size and improve survival [142,143]. These results highlight the possible role of extracellular vesicles in iCCA immunotherapy approaches and may pave the way for the creation of new treatments that specifically target these molecular messengers.

#### 2.2.4. Platelet-Derived Growth Factor

Angiogenesis, fibrosis, and tumor growth are all significantly aided by PDGF [144,145]. Being a strong mitogen, it increases the fibroblast and smooth muscle cell proliferation in peripheral support tissues, which improves the vascular density and structural integrity of tumors [146]. According to recent research, PDGF stimulates tumor cells directly and through signaling pathways like PI3K/Akt and MAPK to increase tumor cell survival and proliferation [147,148]. Furthermore, there is a strong correlation between the prognosis of iCCA patients and high levels of PDGF in TIME; higher levels generally correspond to worse survival and disease progression [149]. As a result, efforts are being made to develop targeted therapeutic approaches against PDGF and its receptors in order to block the growth factor’s pathway, inhibit tumor growth signals, and investigate novel treatment options for iCCA [150,151].

One of these strategies is the application of particular inhibitors, such as imatinib, a small-molecule drug that effectively inhibits the PDGF receptor and has demonstrated potential efficacy in clinical trials for a number of cancers [152,153].

#### 2.2.5. Notch Signaling Pathway

A number of experimental studies suggest a central role for Notch signaling in cholangiocarcinogenesis, acting on tumor cell proliferation, survival, and migration [154]. The canonical Notch signaling pathway in iCCA is known to be a key player in direct cell-cell interactions between cancer cells and the surrounding stromal cells of the microenvironment [155] and has also been shown to be essential for modulating angiogenic sprouting and blood vessel growth in cooperation with VEGFA. Indeed, it has been shown that Crenigacestat, a selective NOTCH1 inhibitor, reduces tumorigenesis in vivo by downregulating the gene expression and protein levels of VEGFA and matrix metalloproteinase-13 (MMP13) [35]. In this context, further studies were conducted in patient-derived xenografts (PDX), and transcriptomic analysis demonstrated that in Crenigacestat-treated mice there was a downregulation of cluster differentiation 90 (CD90), a critical regulator of cell adhesion, communication, and cell-ECM interaction [37]. An intriguing relationship was found between Notch and CD90, which is closely associated with poor prognosis and shorter survival in iCCA patients. Thus, in a subset of patients with iCCA, CD90 is a potential molecular target for treatment. This is the first demonstrated evidence for molecular stratification of iCCA to predict clinical outcomes. [156]. In addition, it has recently been reported that the Notch signaling pathway also interacts with TGF-β in cholangiocarcinogenesis. Specifically, Crenigacestat treatment of patient-derived CAFs inhibits both Notch and TGF-β signaling. This inhibition led to the inactivation of CAFs, resulting in a decrease in the secretion of ECM proteins [157]. Overall, these works showed that inhibition of Notch signaling is effective at two levels, not only reducing tumorigenesis and angiogenesis but also restoring homeostasis in the microenvironment surrounding iCCA with a reduction of desmoplastic reaction [158]. This suggests a potential new strategy to fight tumor progression. A Notch inhibitor, which reduces the desmoplastic reaction and angiogenesis, could be combined with other drugs, such as immune checkpoint inhibitors, which activate the immune response, to increase their effectiveness.

## 3. Immune Evasion and Immunophenotyping in Intrahepatic Cholangiocarcinoma

**Figure 2:**
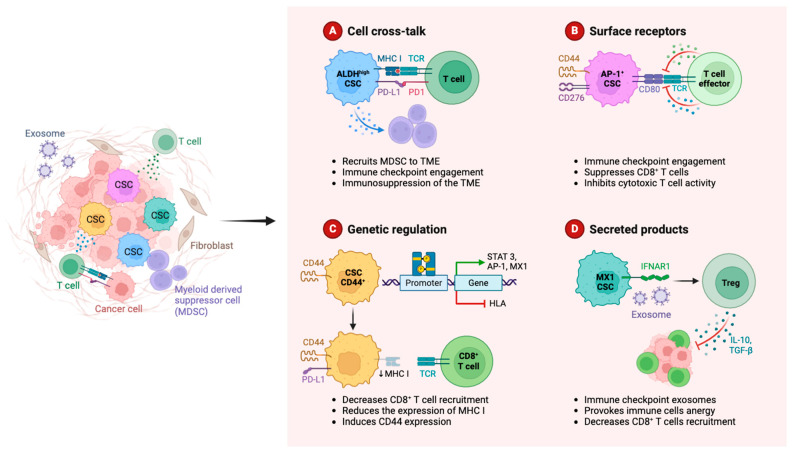
Overview of some mechanisms of immune evasion in cancer.

Tumor cells can avoid immune monitoring and elimination by using an intricate network of immune escape mechanisms, which is employed by iCCA (Figure 2) [159]. The tumor cells of ICCA specifically work to thwart immune attacks by modifying surface antigens and increasing the levels of immunosuppressive molecules [160]. PD-L1, for example, is highly expressed in iCCA cells and binds to PD-1 on T cells to prevent T cell activation and suppress immune responses. In addition, TILs have been shown to express higher levels of CTLA-4, a protein that is linked to aggressive tumor behavior and a poor prognosis [161]. In addition to interfering with metabolic and cell signaling pathways, the expression of these checkpoint molecules also promotes immune tolerance and depletes peripheral T cells.

The interaction between tumor cells and immunosuppressive cells such as MDSCs and TAMs strengthens the immune escape mechanisms within TIME. By releasing inhibitory molecules like arginase and inducible nitric oxide synthase (iNOS), these cells block T cell metabolism and signaling. Furthermore, research suggests that increased PD-L1 expression in iCCA is associated with worse clinical outcomes, such as shorter survival [162]. In approximately 45% of iCCA samples, for example, immune checkpoint molecules were found to be upregulated, indicating a worse prognosis, according to a report published by Nakamura and colleagues [163]. Further investigation has revealed that one of the most important mechanisms for immune escape in iCCA is the control of PD-1 and PD-L1. For example, a study by Gani et al. found that overall survival was 60% lower in 72% of iCCA patients with PD-L1 expression than in those without PD-L1 expression [164]. Furthermore, poorer survival and lymph node metastasis were associated with FOXP3 overexpression in iCCA cells, which is frequently associated with elevated CTLA-4 levels. These findings highlight the detrimental prognostic effect of CTLA-4 in iCCA and highlight the critical function of immune checkpoint molecules in the immune escape mechanism of iCCA.

The relationship between the immunophenotype of a tumor and its biological behavior as well as prognosis has been increasingly clear from studies on immune subtypes [165]. Gene expression profiling has recently led to the classification of iCCA into four immune subtypes: immune-indifferent (I1), with very low levels of immune cell infiltration; immune-activated (I2), marked by substantial infiltration of CD4+, CD8+, and CD45RO+ lymphocytes; myeloid-derived (I3), primarily made up of monocytes and macrophages; and mesenchymal (I4), which is characterized by minimal immune infiltration but significant activation of macrophages and monocytes [166]. The immune-activated subtype (I2) is the most promising among them, showing great promise for immunotherapy and a comparatively better prognosis. Because of this classification, iCCA patients may be eligible for customized immunotherapy based on their unique immune profiles [167]. The molecular immunophenotypes of iCCA have also been further improved by multi-omics-based investigations. In a large-sample genome sequencing study, for example, iCCA was classified into two types: proliferative, which indicates a worse prognosis and is characterized by increased activity in tumor proliferation signaling pathways; and inflammatory, which is characterized by enriched immune signals like interleukins and chemokines [168].

These results provide a theoretical framework for more specialized therapies catered to the various molecular subtypes of iCCA [169,170]. Based on protein expression, recent proteomic analyses identified molecular types, which include inflammatory (S1), mesenchymal (S2), metabolic (S3), and differentiated (S4). Determining these subtypes facilitates accurate tumor diagnosis and treatment, and these studies show that combining data from immunology and molecular biology can improve our knowledge of the biology of iCCA and make it easier to create individualized treatment regimens for individual patients.

## 4. Implications for Treatment and Future Research Avenues

High-throughput sequencing technology has made significant advancements in iCCA molecular typing in recent years. Several classification techniques based on gene mutations (e.g., *IDH1/2*, *FGFR2*, *TP53*), gene expression profiles, and epigenetic modifications are commonly used in molecular typing of iCCA [171]. Research has demonstrated, for instance, that iCCA with *IDH1/2* mutations has particular metabolic characteristics and a different TIME, and similarly, *FGFR2* fusion gene-positive iCCA shows unique growth patterns and drug response [172]. Based on factors such as immunosuppressive molecule expression, cytokine secretion, and the extent of immune cell infiltration in the tumor tissue, the immune microenvironment can be broadly categorized as “inflammatory” or “non-inflammatory” [173,174].

A non-inflammatory TIME is characterized by enhanced immune escape mechanisms, such as high PD-L1 expression and T-cell depletion, whereas an inflammatory TIME is typically characterized by high levels of T-cell infiltration and immunoreactivity. Certain immune microenvironmental phenotypes are closely linked to distinct molecular subtypes of iCCA [175]. For instance, *IDH1/2* mutant iCCA is frequently associated with increased expression of immunosuppressive molecules and decreased T-cell infiltration; iCCA with the *FGFR2* fusion gene, on the other hand, exhibits greater levels of immune cell infiltration and might be more amenable to immunotherapy [176,177]. Immunotherapeutic research in iCCA is developing quickly, posing a wide range of research opportunities, and encountering many obstacles. The ongoing investigation of novel immune checkpoints and other immunotherapeutic targets is expected to broaden the range of potential iCCA treatment approaches. Investigating these novel targets holds the potential to enable customized treatment regimens utilizing precision medicine tools like immunomics and genomics. With this method, “cold tumors” could become “hot tumors,” increasing the immune system’s capacity to fight cancer by better stimulating the body’s immune response [177]. Additionally, the focus of iCCA immunotherapy research in the future will be on creating integrative treatment plans. To combat both primary and acquired tumor resistance, these approaches could combine immunotherapy with other treatments like chemotherapy and targeted therapies, since ICIs have been demonstrated to improve treatment efficacy when combined with other antitumor agents [178,179,180,181,182,183]. Immunotherapy provides new hope for treating iCCA and has recently become a central strategy in increasing the prognosis of iCCA patients; however, several issues are to be considered, including the lack of validated predictive biomarkers that could help to identify patient groups that are most likely to benefit from such therapeutic approaches. Future research must focus on the development of novel agents and combinations, but the validation of biomarkers remains an urgent need.

## 5. Conclusions

While immunotherapy offers a new hope for the treatment of iCCA, the lack of validated predictive biomarkers to determine which patient groups stand to gain the most from these treatments remains an issue. Therefore, to improve the accuracy of therapeutic decisions and boost treatment efficacy, future research must focus on the development and validation of biomarkers. Immunotherapy is set to become a key element in improving the prognosis for patients with iCCA thanks to such extensive research efforts aimed at further comprehending the importance of TIME in this setting.
